# Emergency Knowledge Translation, COVID-19 and indoor air: evaluating a virtual ventilation and filtration consultation program for community spaces in Ontario

**DOI:** 10.1186/s12889-024-20151-2

**Published:** 2024-10-01

**Authors:** Amy Katz, Tianyuan Li, LLana James, Pearl Buhariwala, Jo-Ann Osei-Twum, Jeffrey Siegel, Patricia O’Campo

**Affiliations:** 1 MAP, St. Michael’s Hospital, Unity Health Toronto, Toronto, ON Canada; 2 Faculty of Information, University of Toronto, Toronto, ON Canada; 3 Department of Civil and Environmental Engineering, University of Waterloo, Waterloo, ON Canada; 4 Anansi Health & Innovation Consortium, Toronto, Canada; 5 Department of Biomedical and Molecular Sciences and School of Rehabilitation Therapy, Queen’s University, Kingston, ON Canada; 6 Dalla Lana School of Public Health, University of Toronto, Toronto, ON Canada; 7 Department of Civil and Mineral Engineering, University of Toronto, Toronto, ON Canada

**Keywords:** COVID-19, Public health, Indoor air, Ventilation, Filtration, Knowledge translation, Community education, Engineering

## Abstract

**Background:**

An October, 2021 review of Public Health Ontario's COVID-19 guidance for congregate settings such as shelters and long-term care homes demonstrated that this guidance did not include references to ventilation or filtration. In April 2022, an interdisciplinary team with expertise in indoor air quality (IAQ), engineering, epidemiology, community programming and knowledge translation launched a virtual ventilation and filtration consultation program for community spaces in Toronto, Ontario. The program gives people working in community spaces direct access to IAQ experts through 25-min online appointments. The program aims to help reduce the risk of COVID-19 transmission in community spaces, and was designed to help compensate for gaps in public health guidance and action.

**Methods:**

Representatives from participating organizations (n. 27) received a link to an online survey via email in April 2023. Survey questions explored the impacts of the program on topics such as: purchase and use of portable air filters; maintenance and use of bathroom fans; and, maintenance and modification of HVAC systems. Survey participation was anonymous, and no demographic information was collected from participants.

**Results:**

Representatives from 11 organizations completed the survey (40%). Of those who responded, nine (82%) made changes as a result of the program, with eight (73%) making two or more changes such as purchasing portable air filters and increasing routine maintenance of HVAC systems.

**Conclusions:**

When presented with brief access to expert support and tailored plain language guidance, people working in community spaces increased their use of ventilation and filtration strategies for COVID-19 infection prevention and control.

**Supplementary Information:**

The online version contains supplementary material available at 10.1186/s12889-024-20151-2.

## Background

At the beginning of the COVID-19 pandemic, scientists around the world urged public health authorities such as the World Health Organization and the US Centers for Disease Control and Prevention to take action on airborne transmission [1–3]. These public health authorities did not react immediately to these warnings, however, or to the rapidly evolving evidence that COVID-19 spread through the air [4–6].

This dynamic also played out in Ontario, Canada,^1^ where Public Health Ontario (PHO), an organization mandated by legislation to provide scientific advice during infectious disease outbreaks, did not promote airborne mitigations such as respirator masks and ventilation to all sectors, while scientists and health care providers pushed for change through public communications and the courts [7–9]. An October 2021 study demonstrated that PHO omitted ventilation and filtration from its public, written COVID-19 guidance for long-term care homes and congregate settings such as shelters until June, 2022 [10].

Congregate settings in Ontario suffered devastating consequences from COVID-19 that continue well into the pandemic. For example, according to provincial statistics, between September 2021 and July 2022, 764 LTC residents died of COVID-19 [11]. While there are limited data sets that provide both up-to-date death and outbreak data for other congregate settings in Ontario, updates from local public health agencies [12], reports from media [13, 14], and the work of independent and academic researchers [15] highlight the impact of COVID-19 experienced by people living and working in settings such as group homes, shelters and detention centres.

In the face of the above, our interdisciplinary team made up of experts in community programming and services, indoor air quality (IAQ), engineering, Knowledge Translation (KT), epidemiology and public health set out to make IAQ expertise accessible to community spaces in Toronto, where we live and work. To accomplish this, we implemented a set of linked KT interventions focused on IAQ measures such as ventilation and filtration, which have the capacity to reduce the risk of transmission of respiratory infections, including COVID-19 [16–21]. These interventions have included plain language written guidance [22], webinars, online meetings with representatives of different facilities (e.g. shelters) and a virtual “ventilation and filtration consultation program.”

In this paper, we describe and evaluate the virtual consultation program, which we are working to adjust and improve. In doing so, we demonstrate one component of an iterative “emergency KT” program designed to provide people with access to expert support and guidance.

## Methods

### Program description

#### Goal

In April 2022, the intervention team launched the virtual consultation program. The primary goal of the program is to equip people working in community spaces with actionable information on reducing COVID-19 transmission risk using ventilation and filtration.

#### Program team

The program team consists of: two IAQ experts with PhDs in engineering and advanced training in issues related to IAQ such as filtration, ventilation and Heating, Ventilation and Air-conditioning (HVAC) systems (see expert CVs, SI1); one KT specialist; and, a research manager with training in project management. Both the KT specialist and research manager have extensive experience with group facilitation.

This program team is advised by a broader project team with expertise in KT, epidemiology, implementation science and public health. All project and program team members are co-authors on this paper. In addition to the academic and research expertise outlined above, members of both the project and program team have relationships with and affiliations to local grassroots groups; frontline and management experience in the local community services sector; and, experience working with local community organizations in the context of research initiatives. This knowledge of local context and experience with community programs and services shapes the team's approach to both outreach and group facilitation.

Importantly, the program and project team was advised, inspired and supported over a period of years by a network of research staff and people from community organizations and grassroots groups who shared the ventilation and filtration virtual consultation program with their networks.

#### Defining community spaces

The program was motivated by two reviews of local COVID-19 guidance conducted by our team in fall, 2021. The first review revealed that public, written COVID-19 resources recommended by Toronto Public Health for congregate settings included, "almost no guidance related to ventilation, HVAC systems, portable air filtration or ultraviolet disinfection" ([23], p. 1). The second review revealed that public, written COVID-19 guidance produced by PHO for congregate settings included no references to IAQ measures at all [10]. PHO's COVID-19 guidance at that time for settings such as schools and summer camps, however, *did* include references to ventilation [10]. As a result, the team prioritized congregate settings over settings such as schools. Members of the project team were also aware that different types of community spaces required guidance on using IAQ measures to reduce the risk of COVID-19 transmission. For example, one IAQ expert on our team frequently answered inquiries from the public, including people concerned about community spaces. In addition, multiple types of organizations attended webinars and online meetings about IAQ and COVID-19. Finally, to our knowledge, there was no high quality public, written provincial guidance specifically designed for community spaces such as community centres or drop-ins during the study period. As a result, the program is not limited to congregate settings, but offered to all “community spaces”—loosely defined as spaces and services that are run on a public or non-profit basis.

#### Promotion

The program is advertised as, “a free, 25-min, online consultation with indoor air quality experts from University of Toronto and University of Waterloo.” Anyone working at a community space can book an appointment over email. During periods of promotional pushes, the program was promoted in collaboration with research staff and people from community organizations and grassroots groups, largely over email and, to a lesser degree, social media and through webinars and online meetings. Invitations and flyers (see SI2) were distributed to email listservs, coalitions and networks that include: shelters, drop-ins and community food centres (specifically, those reached by communications from the Toronto Drop-In Network at the time of the study) and comprehensive primary health care organizations (specifically, those reached by the Alliance for Healthier Communities at the time of the study) [24, 25]. These communications likely reached many if not most organizations in these categories in Toronto. Beyond these categories of organization, outreach was largely ad hoc, and focused on the team's networks, and the project email list, which includes workers from community organizations who attended a related webinar and/or the virtual consultation program. The extensive local experience and relationships on the project team helped to ensure the invitations reached many organizations in the types of facilities targeted. Promotional strategies were limited, however, by the emergency nature of the program, and the networks of the team are not comprehensive (see limitations).

#### Appointment structure

Before the appointment, the project manager sends attendees a short list of questions about the space(s) and building(s) they work in. Attendees can share specific questions, along with photos, videos or building plans. The IAQ team reviews this information before the session.

Appointments are held virtually online, and attended by one or both IAQ experts who provide advice, and the KT specialist and research manager who assist with facilitation and follow-up communications. Only one organization is represented per session, although some organizations send multiple attendees.

Following introductions, attendees are invited to ask questions. Generally, attendees ask about the building that houses their organization. In some cases, attendees ask about multiple facilities run by the same organization. Often, attendees have questions about a range of settings within a single facility, for example: a commercial kitchen and a drop-in space; a swimming pool and an activity room; a sleeping area and a staff break room; a clinic area and a waiting room; shared offices and program areas.

#### Consultation content

Where possible, IAQ experts provide advice tailored to specific activities, building conditions and budgets. For example, consultations have included providing advice on reducing transmission in specialized settings such as refrigerated areas, dining rooms and pools. In addition, consultations have addressed facilities with severe budget constraints, inoperable windows, inadequate HVAC systems or no HVAC systems at all.

IAQ experts also share general recommendations to maximize the benefits of filtration and ventilation including: using portable air filters to enhance filtration (except in bathrooms); using bathroom fans that exhaust to the outside; and, opening windows and exterior doors when safety and weather allow. In spaces with centralized HVAC systems, additional general recommendations include working with an HVAC expert to: use the highest efficiency HVAC filter the system can accommodate (ideally with a Minimum Efficiency Reporting Value or "MERV" of 13 or higher; these filters are more efficient at capturing the small particles that may contain viruses); ensure there is a good seal around HVAC filter(s); bring in outdoor air through the HVAC system; and, where the previous conditions have been met, run the system continuously while the building is in operation.

In some contexts, recommendations have included upper-room ultraviolet disinfection, but this is contingent on factors including specific room conditions (e.g. ceiling height) and the availability of experts for design and installation. In keeping with recommendations from the American Society of Heating, Refrigeration and Air-Conditioning Engineers and the Ontario Society of Professional Engineers, the team does not recommend other types of air cleaning technologies [26, 27].

While the team always recommends the use of respirator-grade masks such as N95s or KN95s, mask mandates are not currently in place in Ontario, and each community space has its own masking policy. As a result, IAQ recommendations are shared with the understanding that many if not most community spaces will present non-negligible transmission risks for building occupants while COVID-19 is circulating in the community. The team does recommend, however, identifying spaces and activities that pose a particularly severe risk of transmission, and targeting these for enhanced IAQ measures. Examples include shared dining rooms and bedrooms as well as activities such as singing and fitness [28].

#### Session follow-up

Following each appointment, the research manager sends attendees customized letters over email, along with plain language materials developed by the team [22]. These letters and materials are designed to support attendees to take concrete steps to improve IAQ. The letters are based on the discussion during the appointment, and act as a summary of recommended actions. The letters also provide attendees with a formal communication with the potential to support others to work with them to take action. For example, the letters are designed to be used for funding applications and as tools to encourage decision-makers to implement specific measures. The letters are also intended to clarify and reinforce information shared during the appointments; highlight the most relevant measures based on information shared during the appointment; and ensure there are not misunderstandings. The team is also available for follow-up questions or appointments.

#### Protocols

The team developed protocols to ensure that organizations and individuals would not be penalized for seeking IAQ and occupational health and safety advice, or talking about the realities they face in their facilities. For example, names of attendees and organizations are not shared outside the project team. In addition, the research manager only contacts people who both register for and attend the session with follow-up communication via the email addresses they provide.

In the context of the program’s current protocols, the research manager advises attendees over email that the information they share during the appointment may be used for research purposes, including in public writing, but that this information will not be shared in a way that would allow for identification of individuals or specific organizations. For example, in the context of this evaluation, where an organization offers a relatively unique service, they are categorized in a broad category such as “multi-use community space.”

The team also has protocols around appointment lead-up, structure and facilitation. For example, the team works to set expectations in advance and during the session through the flyer, registration emails and session introduction. Some team members have experience with group facilitation, which has proven important, in particular when there are multiple attendees at the same session. In addition, the team aims to create a warm and respectful atmosphere, where all questions are welcome.

Finally, the team outlines the boundaries of its expertise, emphasizing the importance of on-site advice from licenced professionals, such as HVAC experts, and declining to provide recommendations in response to questions that are out of scope. In addition, the team does not endorse any particular brand of product or particular private consultant in the context of the appointment.

#### Attendance

The appointment is designed to share information with people who are interested in learning about and applying IAQ guidance, and this is how we defined a completed appointment. Thirty-three organizations attended sessions between April 2022 and February 2023. Three organizations, however, were excluded from the evaluation, as attendees did not complete the session. These appointments were not counted as official sessions. These experiences, however, helped the team develop better protocols to screen participant interest and set expectations in advance.

Between April 2022 and February 2023, the team conducted 31 completed appointments with 30 organizations in the Greater Toronto Area—one organization per appointment, with one organization returning for a second appointment. See Table 1, "Types of organizations/spaces that completed virtual consultations, April 2022 – February 2023."

**Table 1:** Types of organizations/spaces that completed virtual consultations, April 2022 February 2023

- Multi-use community spaces (n. 10)
- Congregate living settings such as shelters or supportive housing (n. 6)
- Community health centres or community clinics (n. 5)
- Drop-ins (n. 5)
- Office space for non-profit organizations (n. 4)
Total: 30

Where organizations offered multiple services, they are listed here according to the type of service or services they focused on during the consultation. For example, if an organization was responsible for both drop-in and supportive housing services, they are listed here according to the type of service discussed

As the pandemic was implicitly or explicitly declared over by public health authorities and governments by early 2023, uptake for virtual sessions slowed down, and the team ceased formally promoting the program The intervention team began offering virtual IAQ consultations on an ad hoc basis instead of setting appointment hours in March 2023.

### Evaluation approach

Our evaluation focused on outcomes including material changes (e.g. equipment purchase or retrofit) and practice changes (modified use of existing equipment) with the potential to improve IAQ [29, 30]. We sent an anonymous online survey with a limited number of largely quantitative questions to the email addresses provided to us by people who registered for and attended the program. We chose an online, retrospective survey that could be completed in under 10 min in order to minimize the data collection burden on participants, all of whom worked at community organizations, often in frontline roles. We also chose this approach to ensure continued anonymity for people who participated in the consultation.

### Ethics approval

This evaluation was approved by the “Review of Quality Improvement Studies” (ReQuiST) process at Unity Health Toronto (UHT) on December 20 2022, as it focuses on evaluating and improving an existing program. UHT is an academic hospital system fully-affiliated with the University of Toronto. Approval was received after both program and evaluation details were assessed by ReQuiST for the project application titled: *Indoor Air Quality, COVID-19 and community spaces: Direct Knowledge Translation Program_12_06_22*. Approval was supplied in the form of an email to the program project manager which specified the following: *This initiative was formally reviewed by institutional authorities at Unity Health Toronto and deemed to neither require approval from its Research Ethics Board nor written informed consent from Unity Health Toronto participants*.

### Data collection

All data for this study was collected through an online survey sent to attendees. The project manager sent all attendees who registered for and attended the appointments a cover email containing a link to an anonymous, online survey on April 3, 2023, with one exception (an attendee who had already left their workplace; since this information was known to the evaluation team, the project manager did not attempt to contact this attendee). People also received two follow-up emails (April 17 and May 8). In the end, surveys reached attendees from 27 of the 30 participating organizations. Two emails bounced back as two staff members had left their positions, in both cases these were the only staff members who attended the session for their organization.

Participants were informed of the terms of participation in the introduction to the online survey. They were told that their participation was voluntary and anonymous. They were also told that the team might use anonymized information from the survey in an academic publication. Finally, participants were informed that they were free to skip questions, end the survey at any time, or withdraw their answers by declining to click “submit,” and that their consent to participate was implied if they chose to take part in the survey once they had read the introduction.

Surveys did not collect demographic information on participants. The only information collected related to participants themselves were the following yes/no questions: “Is facility management the primary focus on your role?” and “Is infection prevention and control the primary focus of your role?” Surveys were focused on the outcomes of the ventilation and filtration virtual consultation program in the *physical space of facilities in question*. Participants were asked questions about topics such as their post-session: purchase and use of portable air filters; maintenance and use of bathroom fans; maintenance and modification of HVAC systems; and, adoption of new practices such as clearing the air in rooms between appointments and groups. For the full list of survey questions reported on in this study, see SI3.

Survey questions were brief, and largely quantitative. We assumed participants would not need additional explanation or background materials related to survey questions. We made this assumption as each participant who received the survey had completed a virtual consultation with indoor air quality experts during which all or most options mentioned in the survey questions would have been explored. In addition, all participants received a detailed, plain language letter going over IAQ recommendations. Finally, all participants received plain language guidance developed by the research team, a later version of which was developed in response to questions received during consultations [22].

## Results

Attendees representing 11 of the 27 organizations that received the survey email completed the survey, accounting for 40 per cent of organizations that received the survey and 37 per cent of organizations that completed virtual appointments. In all cases, while the survey was sent to all attendees at each organization, only one attendee from each organization filled out the survey. See Table 2, "Types of organizations/spaces that completed the survey, April 2023." Response rates varied somewhat by organization type (see Table 1 in comparison to Table 2, see also "limitations" for more information re: categorization of organizations). The lowest response rate came from congregate living settings: six attended the consultation and only one completed the survey.

**Table 2:** Types of organizations/spaces that completed the survey, April 2023

- Multi-use community spaces (n. 3)
- Congregate living settings such as shelters or supportive housing (n. 1)
- Community health centres or community clinics (n. 3)
- Drop-ins (n. 2)
- Office space for non-profit organizations (n. 2)
Total: 11

Please note, as the surveys were anonymous, we don’t know if respondents categorized their organizations in the same way that we did

Of the 11 organizations that responded to the survey, nine took concrete steps to improve indoor air quality and/or strengthen masking protocols in their facilities following the session. All concrete steps that were taken by 18 per cent or more participants (two or more organizations) are shown in Table 3, "Concrete, evidence-based IAQ interventions undertaken by two or more survey participants." Actions taken by 35 per cent or more of participants (4 or more organizations) in response to the consultation included: creating or maintaining mask policies; purchase of additional portable air filters; increased use of portable air filters; running bathroom fans continuously when bathrooms were in use; increased routine maintenance of HVAC systems; and, identifying and taking specific measures in higher-risk spaces (e.g. due to occupancy rate or types of activity).

**Table 3:** Concrete, evidence-based IAQ interventions undertaken by two or more survey participants

Evidence-based interventions to reduce risk of airborne transmission of COVID-19^a^	Concrete actions undertaken^b^	Number of survey participants who took this action (11 participants in total took part in the survey)
*Portable air filters*	Purchased additional portable air filters	55% (6)
	Increased use of portable air filters (i.e. used more often, or on a higher setting)	45% (5)
	Change in maintenance of portable air filters (e.g., wearing PPE while changing filters; checking the status of the filters regularly)	36% (4)
	Change in use of portable air filters (e.g., bringing several into a meeting room while a big meeting is going on; positioning portable air filters differently; running multiple on lower settings to reduce noise)	27% (3)
*HVAC systems*^*c*^	Increased routine maintenance, including but not limited to filter changes	36% (4)
	Installing, upgrading or maintaining bathroom fan that exhausts to the outside	18% (2)
	Increasing the amount of outdoor air brought in through HVAC system	18% (2)
*Other practices*	Creating or maintaining masking policies	55% (6)
	Running bathroom fans continuously while bathroom is in use^d^	45% (5)
	Open windows more often	36% (4)
	Identifying and taking specific measures in higher-risk spaces	36% (4)
	Sharing indoor air quality information with staff and other people who use the building	27% (3)
	Cleaning air in the specific rooms after appointments or groups^e^	27% (3)

^a^See "consultation content" section for more information about these interventions. For additional detail, please see plain language checklist developed by our team [22]

^b^This table includes actions taken by two or more survey participants. Actions that were not taken or taken by only one participant are not listed here, please see SI3 for full list of questions and actions

^c^Consultation participants were advised to make these changes in consultation with an HVAC expert

^d^Bathroom fans should exhaust to the outside

^e^Using HVAC systems, portable air filters and/or open windows

Notably, of the nine organizations that took concrete steps, eight undertook at least two measures, such as both purchasing indoor air filters and increasing routine maintenance of HVAC systems. Of the two organizations that did not take concrete steps as a result of the session, one specified that they would be using the information and materials provided by the program to apply for funding to improve IAQ, and the other shared that they presented the recommended action to decision-makers in the organization, who declined to follow this recommended action.

## Discussion

When presented with accessible expert information and brief expert support, people working in community spaces took concrete steps to implement or augment evidence-based IAQ measures that help reduce the risk of COVID-19 transmission.

The most common building-level actions resulting from sessions included: new purchases and/or increased use of portable air filters; increased use and/or maintenance of bathroom fans; and, increased routine maintenance of HVAC systems. More than half of the organizations that filled out the survey also maintained or strengthened masking protocols in response to the session.

Notably, only one organization that filled out the survey upgraded their HVAC filter, and no organizations improved the seal on their HVAC filter slot. The intervention team will explore barriers to these particular actions, and emphasize these actions in future sessions.

The virtual ventilation and filtration consultation program reflects goals and values embedded in public health legislation in our local context such as preventing the spread of infectious disease and working towards health equity (for example, ensuring that all groups of facilities and their workers, visitors and residents receive the best available public health guidance) [9]. It also reflects goals and values articulated more generally in public health theory and practice, for example: addressing infectious disease prevention and control in local context [31]; ensuring that high quality evidence is made accessible to people in a position to apply that evidence directly to improving the "good of the people's health" [32]; and, taking precautionary action while considering the possible harms that could be embedded in any intervention [33].^2^

It is possible the program made a longer-term contribution toward some of these goals, given that the facilities that consulted with us may continue with the IAQ changes made as a result of the consultation, including in the face of future pandemics. We also hope that some of the facilities we reached through the consultation program and webinars continue to consult with us over time, and we have made it clear that our team is always available for questions via email, or for further virtual consultation. Finally, the program contributed to the development of plain language guidance that answers frequently-asked-questions from community spaces, guidance that was not provided by public health authorities in our local context at that time; this guidance was accessed more than 5,700 times within a month of its launch [22].

The program, however, was unable to address the systemic barriers community spaces face when trying to improve IAQ (see “limitations of program”), including the lack of clear, accessible public health guidance around IAQ measures at the time of the study, which was cited by some as a key barrier.

For example, some session participants referenced the need for a document they could share with decision-makers to demonstrate that specific IAQ measures were considered COVID-19 “best practices” by public health authorities. Some participants also expressed concern related to the effectiveness of portable air filters, with at least one attributing this concern to inconsistent public health messaging.

## Strengths, limitations, boundaries and context

### Boundaries of program

While the program has achieved some success, it was time-limited and boundaried.

First, we only connected with a limited number of local community spaces through what was largely an ad hoc, unfunded,^3^ emergency process. If a similar, long-term program were to be instituted in response to future public health emergencies, outreach and attendance should be mapped to ensure comprehensiveness from a range of perspectives. Importantly, there are categories of organization we did not reach. Some, such as detention centres, we did not attempt to reach. More generally, no city-run community spaces attended the program, and very few congregate settings beyond shelters participated.

Second, we do not have the capacity to offer on-site consultations, which emerged as a limitation in some but not all cases. For example, some people had questions about the selection and application of specific IAQ technologies such as portable air filters. Others wanted a list of questions they could share with HVAC consultants. In these cases, site visits would not necessarily have made a big difference. In other cases, however, people asked us to recommend consultants who could come onsite to conduct indoor air quality testing; evaluate performance of HVAC systems; or diagnose building issues.

Our experiences suggest the strong need for access to publicly-funded building performance evaluation. This publicly-funded building performance program should be offered in a timely fashion at no cost to facilities; include a range of multi-disciplinary and applied expertise; and, have the capacity to perform site visits. Through this program, facilities would have access to unbiased assessments, plain language advice, practical next steps and ongoing capacity building. Importantly, any such program should be distinctly siloed from inspection and enforcement. Finally, this type of program could potentially benefit from longer-term monitoring and assessment by building performance experts, such as receiving updated HVAC inspection reports. Any monitoring, however, would need to be done in a way that did not affect occupant privacy.

Finally, our program was unable to address the systemic barriers faced by facility staff who wanted to improve their IAQ such as lack of: comprehensive provincial public health guidance; ventilation requirements for older buildings; long-term or sustainable funding for IAQ infrastructure; and, onsite HVAC and building performance expertise. In some cases, lack of control over building conditions also emerged as an issue. Some of these barriers have also been reported in similar settings such as nursing homes in the US [34].

### Strengths, limitations and notes for evaluation

The findings from this evaluation indicate that the program is meeting its primary goal: to help attendees increase the use of IAQ strategies to reduce COVID-19 transmission in community spaces. As a result, we have decided to offer the program indefinitely, on a per-request basis.

In addition, this evaluation indicates that very few attendees made changes to filter efficiency. As result, in future appointments and plain language documents, we will spend more time discussion how HVAC systems may be adapted to accommodate higher-efficiency filters, and exploring the different factors that affect filter pressure drop.

Several factors may limit the generalizability of our findings to all community spaces.

First, it is possible our findings are not generalizable to all types of congregate or community spaces. In particular they may not be generalizable to categories of organization that we did not complete appointments with such as detention centres, group homes or long-term care homes. It is also possible our findings are not all generalizable outside of our local context.

Second, most people who participated in the program were highly-motivated to implement expert IAQ advice. Our findings may have been different had the program been mandatory, or incentivized by funders or accreditation agencies.

Third, it is likely that people who took action following the appointment were more likely to complete the survey.

Fourth, the vast majority of appointments captured by this study took place at a time of relatively high general concern about COVID-19. Our findings do not reflect program uptake or outcomes at what is currently being characterized as an “endemic” stage of COVID-19.

It should be noted that there may be differences in how the research team categorized respondent organizations, and how survey participants did so. The survey was anonymous, so the research team does not know if participants classified their organization in the same way that we did (e.g. as drop-in or multi-use community space). As a result, and given the small sample size and the fact that we used broad organizational categories to protect the anonymity of participants, response rates by most organization type may not be particularly meaningful.

Finally, we have provided a rapid evaluation of a largely unfunded and relative brief emergency intervention. The intensive period of the intervention lasted just under a year, from April 2022 to February 2023, while concern about COVID-19 was still relatively high. As noted in program limitations, if a similar, long-term program were to be instituted in response to future public health emergencies, outreach and attendance should be mapped to ensure comprehensiveness from a range of perspectives. This would likely produce a larger sample, and allow for more extensive statistical analysis.

## Conclusion

Most people who attended appointments were extremely motivated to implement appropriate mitigation measures for airborne transmission of COVID-19. Some frontline workers in particular had gone to incredible lengths to learn about how to mitigate airborne transmission. Many times people identified some or even most best practices through their own research. Occasionally, people concluded from their own research that evidence-based interventions such as portable air filters may not be helpful.

In all cases, people were attempting to reduce the risk of COVID-19 transmission in the absence of readily-accessible high quality provincial public health guidance for their facilities, and out of concern for health and safety. The project team continues to be inspired by the commitment, skill, creativity and determination of people attempting to keep themselves and others as safe as they possibly can.

In our experience, people are motivated to protect their own health and safety, and the health and safety of the people who visit, live in and work in community spaces and facilities. While people who attended appointments identified a range of barriers to using ventilation and filtration to reduce transmission of COVID-19 (e.g. funding), many took additional action when presented with accessible expert information and brief expert support.

## Supplementary Information

Supplementary Material 1.

Supplementary Material 2.

Supplementary Material 3.

## Data Availability

The datasets used for the current study are available from the corresponding author on reasonable request.

## Abbreviations

HVAC: Heating, Ventilation and Air-Conditioning
IAQ: Indoor Air Quality
KT: Knowledge Translation
MERV: Minimum Efficiency Reporting Value
PHO: Public Health Ontario

## Footnotes

1 "Ontario" and "Canada" refer to settler colonial place names, boundaries and governance structures. As stated in a related study by our team, we do not focus on jurisdictions such as Ontario, "…to suggest these are fixed and inevitable categories. Rather, we explore the mandate and actions of [Public Health Ontario] PHO in the context of the current settler colonial matrix of governance that helps to determine the conditions under which many people in what is currently called Ontario work and live" ([7], p692).

2 For a discussion of the potential for precautionary action such as some surveillance and enforcement measures to cause harm, and in particular to "reproduce the dynamics of oppression and advantage in a specific jurisdiction," please see Katz et al. 2023 [7]. In the context of this discussion, we note that IAQ measures are considered best practice in non-pandemic conditions, and can be implemented without stigmatizing, surveilling or limiting the activities of building occupants.

3 The only purpose-funded staff position for both the virtual consultation and the evaluation is the part-time research manager who coordinates outreach, appointments and follow-up letters and helps to facilitate appointments. All other contributions from both IAQ experts and the rest of the program and project team are services offered in-kind.
