# Supplementary material for: Emergency Knowledge Translation, COVID-19 and indoor air: evaluating a virtual ventilation and filtration consultation program for community spaces in Ontario
Source: BMC Public Health. 2024 Oct 1;24:2682. doi: 10.1186/s12889-024-20151-2 (PMC11443783; doi:10.1186/s12889-024-20151-2)
Supplement: Supplementary file 1 — Supplementary Material 1. [file 12889_2024_20151_MOESM1_ESM.pdf]

**Amy (Tianyuan) Li, Ph.D.**
*Assistant Professor, Department of Civil and Environmental Engineering*
*The University of Waterloo, 200 University Avenue West, Waterloo ON*
*Tianyuan.li@uwaterloo.ca*

## EDUCATION

| | | | |
|---|---|---|---|
| The University of Toronto | Department of Civil and Mineral Engineering | Ph.D. | 2021 |
| | | BASc | 2016 |

## PEER-REVIEWED JOURNAL PUBLICATIONS (submitted or published)

12. **Li T**, Wan Y, Diamond ML, Siegel JA. 2023. Quantitative filter forensics for allergens and SVOCs in residential buildings. Submitted to *Building and Environment*.
11. Sparks M, Farahbakhsh I, Anand M, Bauch CT, Conlon KC, East JD, **Li T**, Lickley MJ, Garcia-Menendez F, Monier E, Saari RK. 2023. Interactions of short-term adaptation, climate change mitigation, and climate variability in protecting human health from air pollution. Submitted to *Proceedings of the National Academy of Sciences*.
10. Katz A, **Li T**, James L, Siegel JA, O'Campo P. 2023. Systematically omitting indoor air quality: substandard guidance for shelters, group homes and long-term care during the COVID-19 pandemic. *Critical Public Health*. DOI: 10.1080/09581596.2023.2262736
9. **Li T**, Siegel JA. 2021. The impact of control strategies on filtration performance. *Energy & Buildings*, **251**, 111378. DOI: 10.1016/j.enbuild.2021.111378
8. **Li T**, Siegel JA. 2021. Assessing the impact of filtration systems in indoor environments with effectiveness. *Building and Environment*, **87**, 107389. DOI: 10.1016/j.buildenv.2020.107389
7. Matava C, Collard V, Siegel J, Denning S, **Li T**, Du B, Fiadjoe J, Fiset P, Engelhardt T. 2020. Use of a high-flow extractor to reduce aerosol exposure in tracheal intubation. *British Journal of Anaesthesia*, **125**, E363-366. DOI: 10.1016/j.bja.2020.07.014
6. Zhang Y, **Li T**, Siegel JA. 2020. Investigating the impact of filters on long-term particle concentration measurements in residences (RP-1649). *Science and Technology for the Built Environment*, **26(8)**, 1037-1047. DOI: 10.1080/23744731.2020.1778402
5. **Li T**, Siegel JA. 2020. Laboratory performance of new and used residential HVAC filters: Comparison to field results (RP-1649). *Science and Technology for the Built Environment*, **26(6)**, 844-855. DOI: 10.1080/23744731.2020.1738871 (***Featured in ASHRAE Journal Newsletter***)
4. **Li T**, Siegel JA. 2020. In-situ efficiency of filters in residential central HVAC systems. *Indoor Air*, **30(2)**, 315-325. DOI: 10.1111/ina.12633
3. Alavy M, **Li T**, Siegel JA. 2020. Energy use in residential buildings: Analyses of high-efficiency filters and HVAC fans. *Energy & Buildings*, **209**, Article 109697. DOI: 10.1016/j.enbuild.2019.109697
2. **Li T**, Alavy M, Siegel JA. 2019. Measurement of residential HVAC runtime. *Building and Environment*, **150**, 99-107. DOI: 10.1016/j.buildenv.2019.01.004
1. Alavy M, **Li T**, Siegel JA. 2018. Exploration of a long-term measurement approach for air exchange rate. *Building and Environment*, **144**, 474-481. DOI: 10.1016/j.buildenv.2018.08.051

## REPORTS

2. **Li T**, Katz A, Siegel JA. (2022). *Reducing transmission of COVID-19 through improvements to indoor air quality: a checklist for community spaces*. Unity Health Toronto, University of Toronto, University of Waterloo, Queen's University and Toronto Metropolitan University, Toronto, ON. Oct 2022. Available at: http://maphealth.ca/ventilation
1. **Li T**, Alavy M, Zhang Y, Siegel JA. *IAQ and Energy Implications of High-Efficiency Filters in Residential Buildings (1649-RP).* American Society of Heating, Refrigerating and Air-conditioning Engineers, Atlanta, GA. Dec 2019.

**PEER-REVIEWED CONFERENCE ABSTRACTS/EXTENDED ABSTRACTS**

12. Al Humidi S, **Li T**. 2023. Assessing the impact of poor-performing homes on indoor air quality pre- and post-energy retrofits in low-income populations. Submitted to *ASHRAE Transactions for the 2024 Winter Conference*, Paper 35702.
11. **Li T**. 2023. Quantifying ultrafine particle exposure and the effectiveness of mitigation strategies for 3D printers in various operating environments. *Proceedings of the American Association for Aerosol Research 41st Annual Conference*, Paper 12.CM.2.
10. **Li T**, Wan Y, Du, B, Diamond M, Siegel JA. Qualitative Filter Forensics for Allergens and SVOCs. Indoor Air 2022: *Proceedings of the 16th International Conference on Indoor Air and Climate*.
9. **Li T**, Siegel JA. Novel Control Strategies for Indoor Filtration. *Proceedings of the American Association for Aerosol Research 39th Annual Conference*, Paper 4.IA.3.
8. Du B, **Li T**, Matava C, Collard V, Denning S, Fiadjoe J, Fiset P, Engelhardt T, Siegel JA. Using a High-Flow Extractor to Reduce Aerosol Exposure in Medical Environments**.** *Proceedings of the American Association for Aerosol Research 39th Annual Conference*, Paper 3.IA.9.
7. **Li T**, Siegel JA. The in-situ and laboratory efficiency of new and used residential HVAC filters. Indoor Air 2020: *Proceedings of the 16th International Conference on Indoor Air and Climate*, Paper 0128.
6. **Li T**, Siegel JA. Indoor air quality and energy implications of high-efficiency filters in residential buildings. Indoor Air 2020: *Proceedings of the 16th International Conference on Indoor Air and Climate*, Paper 0130.
5. **Li T**, Alavy M, Zhang Y, Siegel JA. 2020. Does residential HVAC filtration work? *Proceedings of the American Association for Aerosol Research 38th Annual Conference*, Paper 11.IA.3.
4. **Li T**, Siegel JA. Impacts of HVAC sequencing on runtime. Indoor Air 2018: *Proceedings of the 15th International Conference on Indoor Air and Climate*, Paper 645.
3. Alavy M, **Li T**, Siegel JA. Long-term air exchange rates in a residence. Indoor Air 2018: *Proceedings of the 15th International Conference on Indoor Air and Climate*, Paper 751.
2. Alavy M, **Li T**, Mahdavi A, Siegel JA. In-situ integrated filter efficiency measurement. *Proceedings of ASHRAE Houston 2018*.
1. Diaz Lozano Patiño E, **Li T**, Vantresca M, Vera Zambrano M, Xie H. 2017. Characterizing particulate matter emissions from an ultrasonic essential oil diffuser. *Proceedings of Healthy Buildings 2017 Asia*, 329-331.

**EXTERNAL COMMITTEES AND PROFESSIONAL SERVICES**

- Member of the Indoor Air Quality Advisory Group of the Ontario Society of Professional Engineers
- Member of American Society of Heating, Refrigerating and Air Conditioning Engineers (ASHRAE) Technical Committee 2.4

**REVIEW PANELS AND EDITORIAL ROLES**

- Member of the International Scientific Committee for Indoor Air 2020 and 2022
- Proposal review for NSERC Alliance Grant
- Journal review for Building and Environment, Indoor Air, Building Simulations, Journal of Exposure Science and Environmental Epidemiology

**MEMBERSHIP**

- ASHRAE YEA member 2021 - present
- ASHRAE Student member 2017 - 2021
- ISIAQ student member 2017 - 2021

**Jeffrey A. Siegel, Ph.D.**
*Professor, Department of Civil and Mineral Engineering*
*Bahen/Tanenbaum Chair in Civil Engineering*
*The University of Toronto*

---

**EDUCATION**

| | | | |
|---|---|---|---|
| University of California, Berkeley | Mechanical Engineering | Ph.D. | 2002 |
| University of California, Berkeley | Mechanical Engineering | M.S. | 1999 |
| Swarthmore College | Engineering | B.S. | 1995 |

**ACADEMIC EXPERIENCE**

*The University of Toronto, Department of Civil and Mineral Engineering*
Professor – 7/2015 - present
Associate Professor – 1/2013 to 6/2015
*The University of Toronto, School of Public Health (non-budgetary cross appointment)*
Professor – 9/2014 to present
*The University of Toronto Department of Physical and Environmental Sciences (non-budgetary cross appointment)*
Professor – 9/2015 to present
*The University of Texas at Austin, Department of Civil, Architectural, and Environmental Engineering*
Associate Professor – 9/2008 to 12/2012
Assistant Professor – 8/2002 to 8/2008

**COURSES TAUGHT (*University of Toronto*)**
CIV 1320: Indoor Air Quality, CIV 576 Sustainable Buildings, CIV 380 Sustainable Energy Systems CIV 375/575 Building Science

**COURSES TAUGHT (*University of Texas)***
ARE389T: Indoor Air Quality: Transport and Control, ARE370: Design of Energy Efficient and Healthy Buildings, ARE346N Building Environmental Systems, ARE346P: HVAC Design, ARE383 Advanced Sustainable Buildings, CE383 Indoor Environmental Quality Measurements

**GRADUATED STUDENTS *(University of Toronto)*** 6 PhD, 8 MASc/MPH, 2 PDF, 4 in progress

**GRADUATED STUDENTS *(University of Texas)*** 13 PhD,17 MS

**SELECTED AWARDS**
- UTFA Academic Citizen Award (2022)
- Bahen/Tanenbaum Chair in Civil Engineering (2021-)
- ASHRAE Fellow (2017-)
- Member of International Society of Indoor Air Quality and Climate Academy of Fellows (2016-)
- 2010 ASHRAE Transactions Paper Award, with Michael Waring (2011)
- J. Neils Thompson Centennial Teaching Fellow in Civil Engineering (2010-2012)
- Student Engineering Council Departmental Favorite Professor Award (2008)
- College of Engineering Outstanding Teaching by an Assistant Professor (2007)
- ASHRAE New Investigator Award (2006, 2007)
- International Society for Exposure Assessment Early Career Award (2004-2007)

**CURRENT AND RECENT EXTERNALLY FUNDED RESEARCH PROJECTS**
CIHR, NSERC (4), NFRF, Sloan Foundation, MITACS, CFI/ORF

**SELECTED JOURNAL ARTICLES FROM PAST SIX YEARS (*121 Career Total)***

118 Persily AK, Siegel JA. 2022. Improving Ventilation Performance in Response to the Pandemic. *The Bridge* 52(3). https://www.nae.edu/281056/Microbiomes-of-the-Built-Environment-

114 Wan Y, Diamond ML, Siegel JA. 2022. Quantitative Filter Forensics for Semi-volatile Organic Compounds (SVOCs) in Social Housing Apartments. *Indoor Air*, **32(2)**, e12994. DOI: https://doi.org/10.1111/ina.12994

113. Mendell A, Mahdavi A, Siegel JA. 2022. Particulate matter concentrations in social housing. *Sustainable Cities & Society*, **76**, 103503. DOI: 10.1016/j.scs.2021.103503

110. Schwartz-Narbonne H, Abbatt J, DeCarlo PF, Farmer DK, Mattila J, Wang C, Donaldson DJ, Siegel JA. 2021. Modelling the removal of water-soluble trace gases from indoor air via air conditioner condensate. *Environmental Science and Technology*, **55**, 10987-10993. DOI: 10.1021/acs.est.1c02053

107. Maestre JP, Jarma D, Yu J-R F, Siegel JA, Horner S, Kinney KA. 2021. Distribution of SARS-CoV-2 RNA signal in a home with COVID-19 positive occupants. *Science of the Total Environment*, **778**, 106201. DOI: 10.1016/j.scitotenv.2021.146201

104. Mahdavi A, Dingle J, Chan AWH, Siegel JA. 2021. HVAC filtration of particles and trace metals: Airborne measurements and the evaluation of quantitative filter forensics. *Environmental Pollution*, **271**, 116388. DOI: 10.1016/j.envpol.2020.116388

103. Mahdavi A, Siegel JA. 2021. Quantitative filter forensics: Size distribution and particulate matter concentrations in residential buildings. *Indoor Air*, **31(4)**, 1050-1060. DOI: 10.1111/ina.12782

101. Vakalis D, Lepine C, MacLean HL, Siegel JA. 2021. Can green schools influence academic performance? *Critical Reviews in Environmental Science and Technology*, **51(13)**, 1354–1396. DOI: 10.1080/10643389.2020.1753631

100. Rajagopalan S, Brauer B, Bhatnagar A, Bhatt DL, Brook JR, Huang W, Münzel T, Newby D, Siegel JA, Brook RD. 2020. Personal-level protective actions against particulate matter air pollution exposure: A scientific statement from the American heart association. *Circulation*, **142(23)**, e411-431. DOI: 10.1161/CIR.0000000000000931

99. Newman et al. (23 authors). 2020. Cardiopulmonary impact of particulate air pollution in high-risk populations. *Journal of the American College of Cardiology*, **26**, 2878-2894. DOI: 10.1016/j.jacc.2020.10.020

96. Du B, Tandoc M, Mack M, Siegel JA. 2020. Indoor $CO_2$ concentrations and cognitive function: A critical review. *Indoor Air*, **30(6)**, 1067-1082. DOI: 10.1111/ina.12706

95. Mahdavi A, Siegel JA. 2020. Extraction of dust collected in HVAC filters for quantitative filter forensics. *Aerosol Science and Technology*, **54(11)**, 1282-1292. DOI: 10.1111/ina.12782

89. Li T, Siegel JA. 2020. In-situ efficiency of filters in residential central HVAC systems. *Indoor Air*, **30(2)**, 315-325. DOI: 10.1111/ina.12633

84. Li T, Alavy M, Siegel JA. 2019. Measurement of residential HVAC runtime. *Building and Environment*, **150**, 99-107. DOI: 10.1016/j.buildenv.2019.01.004

82. Givehchi R, Maestre JP, Bi C, Wylie G, Xu Y, Kinney K, Siegel JA. 2019. Quantitative filter forensics with residential HVAC filters to assess indoor concentrations. *Indoor Air*, **29(3)**, 390-402. DOI: 10.1111/ina.12536

76. Touchie M, Siegel JA. 2018. Residential HVAC runtime from smart thermostats: Characterization, comparison, and impacts. *Indoor Air*, **28(6)**, 905-915. DOI: 10.1111/ina.12496

73. Maestre JP, Jennings W, Wylie G, Horner S, Siegel JA, Kinney K. 2018. Filter forensics: Microbiome recovery from residential HVAC filters. *Microbiome*, **6**, 22. DOI: 10.1186/s40168-018-0407-6

70. Haaland D, Siegel JA. 2017. Quantitative filter forensics for indoor particle sampling. *Indoor Air*, **27(2)**, 364-376. DOI: 10.1111/ina.12319

66. Siegel JA. 2016. Keynote: Primary and secondary consequences of indoor air cleaners. *Indoor Air*, **26(1)**, 88-96. DOI: 10.1111/ina.12194
