# Supplementary material for: Emergency Knowledge Translation, COVID-19 and indoor air: evaluating a virtual ventilation and filtration consultation program for community spaces in Ontario
Source: BMC Public Health. 2024 Oct 1;24:2682. doi: 10.1186/s12889-024-20151-2 (PMC11443783; doi:10.1186/s12889-024-20151-2)
Supplement: Supplementary file 2 — Supplementary Material 2. [file 12889_2024_20151_MOESM2_ESM.pdf]

Indoor air quality consultations for community spaces and congregate settings

Presented by University of Toronto, University of Waterloo, and the MAP Centre for Urban Health Solutions, with funding from the School of Cities at the University of Toronto.

Do you work at a community space or congregate setting (like a shelter or a group home)? Would you like advice about reducing transmission of COVID-19 through indoor air quality measures like ventilation and filtration? Here's your chance! You can ask questions about HVAC systems, portable air filters, UV disinfection, and more! You can ask questions about particular rooms or your whole building. These sessions are open to anyone working in community spaces including facility managers; people responsible for infection prevention and control; and workers who have questions about how indoor air quality measures can help make workplaces safer for everyone.

What: A free, 25 minute, online consultation with indoor air quality experts from the University of Toronto and the University of Waterloo.

How to register: To schedule an online appointment email: Pearl.Buhariwala@unityhealth.to.

How to prepare: Please explore this checklist. Please also bring as much information as possible. For example, if you have questions about your HVAC system, find out as much about it as you can before the appointment.

About our experts:

Jeff Siegel is a Professor in the Department of Civil and Mineral Engineering at University of Toronto. His research interests include healthy and sustainable buildings, ventilation and indoor air quality.

Amy (Tianyuan) Li is an Assistant Professor in the Department of Civil and Environmental Engineering at University of Waterloo. Her research focuses on the impact of filtration devices on indoor air quality.

Why we're doing this: Indoor air quality measures can help reduce transmission of COVID-19. They should be part of every facility's plan for infection prevention and control. For more about improving indoor air quality, please see here.

Please note: these consultations are offered for informational purposes only, and do not replace or remove the need for on-site advice from licensed professionals. We are not responsible for the way you or your organization apply the information shared during this session. Please also note that we are not engineers or medical professionals.
