# Supplementary material for: Emergency Knowledge Translation, COVID-19 and indoor air: evaluating a virtual ventilation and filtration consultation program for community spaces in Ontario
Source: BMC Public Health. 2024 Oct 1;24:2682. doi: 10.1186/s12889-024-20151-2 (PMC11443783; doi:10.1186/s12889-024-20151-2)
Supplement: Supplementary file 3 — Supplementary Material 3. [file 12889_2024_20151_MOESM3_ESM.pdf]

Supplementary Information, File 3

Emergency Knowledge Translation, COVID-19 and indoor air: evaluating a virtual ventilation and filtration consultation program for community spaces in Ontario

<u>Online survey questions reported on in study</u>*

1. If you are comfortable doing so, please let us know what type of organization you consulted us about:

- Congregate living setting (e.g. shelter or supportive housing)
- Drop-In
- Community health centre or community clinic
- Multi-use community space
- Office space
- Other (Please specify)

2. Did our appointment, follow-up letter and/or plain language guidance lead to one or more of the following actions related to portable air filters? Please select all that apply:

- Purchases of additional portable air filters (conventional store bought portable air filters or DYI box filters)
- Increased use of portable air filters (i.e. used more often, used on a higher setting)
- Change in use of portable air filters (e.g. bringing several into a meeting room while a big meeting is going on; positioning portable air filters differently; running multiple portable air filters on lower settings to reduce noise)
- Change in maintenance of portable air filters (wearing PPE while changing filters; checking the status of the filters regularly)
- Other (Please specify)

3. Did our appointment, follow-up letter and/or plain language guidance lead to one or more of the following actions in consultation with an HVAC contractor or upper-room UV specialist? Please select all that apply:

- Installing, upgrading, or maintaining bathroom fan that exhausts to the outside
- Upgrading of furnace filter in consultation with HVAC contractor (i.e. moving to a higher minimum efficiency reporting value or "MERV")
- Improving seal around HVAC filter(s)
- Increasing the amount of outdoor air brought in through HVAC system
- In cases where the HVAC system provides adequate ventilation and filtration, changing the HVAC fan from "auto" to "on" while the building is in use to ensure continuous ventilation and filtration

- Increasing routine maintenance of HVAC systems, including but not limited to filter changes
- Installing upper-room ultraviolet disinfection
- Other (Please specify)

4. Did our appointment, follow-up letter and/or plain language guidance lead to one or more of the following practices (please do not select practices that were already in place prior to the consultation). Please select all that apply:

- Cleaning the air in specific rooms after appointments or groups
- Running bathroom fans continuously while the bathroom is in use
- Opening windows more often
- Creating or maintaining masking policies
- Identifying and taking specific measures in higher-risk spaces or during higher-risk activities
- Sharing indoor air quality information with staff and other people who use the building
- Discontinuing the use of unproven technologies (e.g. disabling ionization on portable air filters)
- Monitoring indoor CO_2 concentrations
- Other (Please specify)

* The list of measures above is not intended to be used in isolation without further guidance. For context related to the measures listed above, please consult the following document:

Li T, Katz A, Siegel J. Reducing Transmission of COVID-19 Through Improvements to Indoor Air Quality - A checklist for Community Spaces [Internet]. Unity Health Toronto, University of Toronto, University of Waterloo, Queen’s University and Toronto Metropolitan University; 2022 [cited 2023 Feb 5]. Available from: https://maphealth.ca/ventilation/
